# Supplementary material for: Integrated rapid risk assessment for dengue fever in settings with limited diagnostic capacity and uncertain exposure: Development of a methodological framework for Tanzania
Source: PLoS Negl Trop Dis. 2025 Mar 28;19(3):e0012946. doi: 10.1371/journal.pntd.0012946 (PMC11978086; doi:10.1371/journal.pntd.0012946)
Supplement: S1 Text — COMPARTMENT I. (DOCX) [file pntd.0012946.s001.docx]

**S1 Text**

**Identification of indicators relevant to the public health risk assessment for dengue outbreaks**

*COMPARTMENT II*

We identified temperature, precipitation and humidity as key factors in *Aedes aegypti* mosquitoes, which impact all life stages (eggs, larvae, pupae, adult), including reproduction, behavior and survival (1-8). While *Aedes aegypti* can tolerate temperatures ranging from 14-30°C and even survive extremes >40°C (8), the ideal condition for sustaining all life stages is 20-30°C (3, 9). In contrast, *Aedes* larvae are unable to develop at below 10-15°C (10), and as adults they are less mobile and unable to feed below 14-15°C, resulting in their death (3). Ambient temperature is also important for replication and survival of DENV inside the mosquito (2). The optimal temperature is 23-29°C (1). Therefore, we have established that a minimum temperature of >15°C is critical for *Aedes aegypti* survival, and mean temperatures between 23-29°C are optimal for sustained dengue transmission.

In addition, temperature interacts with precipitation and humidity, influencing the hatching of eggs and promoting habitats for the aquatic mosquito life stages in both natural and artificial water sources (3, 8), which significantly impacts vector abundance (11). However, the effects of rain quantity and frequency as well as humidity levels are not well understood, and the influence of rainy and dry seasons is often used as an indicator of conditions conducive to mosquitoes. While the *Aedes* vector can be present in both the rainy and dry seasons, larvae and pupae infestation rises significantly from the dry to the rainy season, leading to a higher risk of high vector density (12, 13). Given this context and historical data from Tanzania that indicate dengue outbreaks are more prevalent in the rainy season (14), we have incorporated seasonality (dry vs. rainy) into our framework to account for variations in precipitation and humidity.

**References**

1. Mordecai EA, Caldwell JM, Grossman MK, Lippi CA, Johnson LR, Neira M, Rohr JR, Ryan SJ, Savage V, Shocket MS, Sippy R, Stewart Ibarra AM, Thomas MB, Villena O. Thermal biology of mosquito-borne disease. Ecol Lett. 2019;22(10):1690-708.

2. Liu Z, Zhang Q, Li L, He J, Guo J, Wang Z, Huang Y, Xi Z, Yuan F, Li Y, Li T. The effect of temperature on dengue virus transmission by Aedes mosquitoes. Front Cell Infect Microbiol. 2023;13:1242173.

3. Alto BW, Bettinardi D. Temperature and dengue virus infection in mosquitoes: independent effects on the immature and adult stages. Am J Trop Med Hyg. 2013;88(3):497-505.

4. Brugueras S, Fernández-Martínez B, Martínez-de la Puente J, Figuerola J, Porro TM, Rius C, Larrauri A, Gómez-Barroso D. Environmental drivers, climate change and emergent diseases transmitted by mosquitoes and their vectors in southern Europe: A systematic review. Environ Res. 2020;191:110038.

5. Robert MA, Stewart-Ibarra AM, Estallo EL. Climate change and viral emergence: evidence from Aedes-borne arboviruses. Curr Opin Virol. 2020;40:41-7.

6. Li Y, Dou Q, Lu Y, Xiang H, Yu X, Liu S. Effects of ambient temperature and precipitation on the risk of dengue fever: A systematic review and updated meta-analysis. Environ Res. 2020;191:110043.

7. Li C, Wang X, Wu X, Liu J, Ji D, Du J. Modeling and projection of dengue fever cases in Guangzhou based on variation of weather factors. Sci Total Environ. 2017;605-606:867-73.

8. OECD. Safety Assessment of Transgenic Organisms in the Environment, Volume 8: OECD Consensus Document of the Biology of Mosquito Aedes aegypti, Harmonisation of Regulatory Oversight in Biotechnology. OECD Publishing; 2018 [cited 2024 May 23]. Available from: <https://doi.org/10.1787/9789264302235-en>.

9. Tun-Lin W, Burkot TR, Kay BH. Effects of temperature and larval diet on development rates and survival of the dengue vector Aedes aegypti in north Queensland, Australia. Med Vet Entomol. 2000;14(1):31-7.

10. Reiskind MH, Zarrabi AA. Is bigger really bigger? Differential responses to temperature in measures of body size of the mosquito, Aedes albopictus. J Insect Physiol. 2012;58(7):911-7.

11. Morin CW, Comrie AC, Ernst K. Climate and dengue transmission: evidence and implications. Environ Health Perspect. 2013;121(11-12):1264-72.

12. Wai KT, Arunachalam N, Tana S, Espino F, Kittayapong P, Abeyewickreme W, Hapangama D, Tyagi BK, Htun PT, Koyadun S, Kroeger A, Sommerfeld J, Petzold M. Estimating dengue vector abundance in the wet and dry season: implications for targeted vector control in urban and peri-urban Asia. Pathog Glob Health. 2012;106(8):436-45.

13. Gómez-Vargas W, Ríos-Tapias PA, Marin-Velásquez K, Giraldo-Gallo E, Segura-Cardona A, Arboleda M. Density of Aedes aegypti and dengue virus transmission risk in two municipalities of Northwestern Antioquia, Colombia. PLoS One. 2024;19(1):e0295317.

14. Lee HS, Nguyen-Viet H, Nam VS, Lee M, Won S, Duc PP, Grace D. Seasonal patterns of dengue fever and associated climate factors in 4 provinces in Vietnam from 1994 to 2013. BMC Infect Dis. 2017;17(1):218.
