# Supplementary material for: Integrated rapid risk assessment for dengue fever in settings with limited diagnostic capacity and uncertain exposure: Development of a methodological framework for Tanzania
Source: PLoS Negl Trop Dis. 2025 Mar 28;19(3):e0012946. doi: 10.1371/journal.pntd.0012946 (PMC11978086; doi:10.1371/journal.pntd.0012946)
Supplement: S2 Text — COMPARTMENT III. (DOCX) [file pntd.0012946.s002.docx]

**S2 Text**

**Identification of indicators relevant to the public health risk assessment for dengue outbreaks**

*COMPARTMENT III*

Our framework operates across two dimensions – (i) factors influencing outbreak progression (21 indicators) and (ii) factors influencing the impact of an outbreak on the population and the economy (7 indicators) – incorporating both disease-specific (dengue) and generally applicable indicators (see Table 2a and 2b in the manuscript):

We identified four clusters of indicators relevant to influence outbreak progression. First, and most relevant, diagnostic and surveillance capacities are needed to detect (dengue) infections and potential outbreaks. There are several studies testing and evaluating early response to epidemic situations, indicating the importance of both rapid diagnostic tests (RDT) (1, 2) and confirmatory diagnostics, as well as availability of laboratory personnel in the country (3). Second, sociodemographic factors such as population (4, 5) and household density (6), along with socioeconomic factors such as education (7) and poverty (5, 6), contribute to the duration and outcome of outbreaks by shaping local vulnerability and resilience, including transmission rates, risk awareness, and compliance with control interventions, among others. In addition, factors like water supply (6), sanitation (8, 9), and garbage collection (10) are taken into account, as they influence mosquito infestation and control efforts. Third, transnational travel and trade activities significantly influence disease distribution through mobility networks, including airports (11, 12), seaports (12), rail and road infrastructure (13). Moreover, the proportion of the mobile (working) population is considered a crucial factor in the regional spread of dengue along their routes (14). Fourth, outbreak progression is influenced by vector-associated factors (15) including control strategies (5), evidence of vector abundance, the adoption of (personal) protective measures such as insecticide-treated nets (7), and environmental conditions favorable to *Aedes* mosquitoes, such as urban environment (4, 8) and suitable weather conditions (16).

Following the same schema, we identified two clusters of indicators that influence the population and economic impact of an outbreak. Overall factors of healthcare capacity in the region include the density of physicians, nurses, and midwives, as well as the number of hospital beds and access to care (travel time to the nearest facility) (17-19). Dengue-specific factors encompass sociodemographic characteristics that shape morbidity and mortality, including vulnerable age groups (20, 21), to account for health and economic costs, such as years of life lost (22).

**References**

1. Li Y, Kamara F, Zhou G, Puthiyakunnon S, Li C, Liu Y, Zhou Y, Yao L, Yan G, Chen XG. Urbanization increases Aedes albopictus larval habitats and accelerates mosquito development and survivorship. PLoS Negl Trop Dis. 2014;8(11):e3301.

2. Alenou LD, Nwane P, Mbakop LR, Piameu M, Ekoko W, Mandeng S, Bikoy EN, Toto JC, Onguina H, Etang J. Burden of mosquito-borne diseases across rural versus urban areas in Cameroon between 2002 and 2021: prospective for community-oriented vector management approaches. Parasit Vectors. 2023;16(1):136.

3. Kolimenakis A, Heinz S, Wilson ML, Winkler V, Yakob L, Michaelakis A, Papachristos D, Richardson C, Horstick O. The role of urbanisation in the spread of Aedes mosquitoes and the diseases they transmit-A systematic review. PLoS Negl Trop Dis. 2021;15(9):e0009631.

4. Vairo F, Nicastri E, Meschi S, Schepisi MS, Paglia MG, Bevilacqua N, Mangi S, Sciarrone MR, Chiappini R, Mohamed J, Racalbuto V, Di Caro A, Capobianchi MR, Ippolito G. Seroprevalence of dengue infection: a cross-sectional survey in mainland Tanzania and on Pemba Island, Zanzibar. Int J Infect Dis. 2012;16(1):e44-6.

5. Vairo F, Mboera LE, De Nardo P, Oriyo NM, Meschi S, Rumisha SF, Colavita F, Mhina A, Carletti F, Mwakapeje E, Capobianchi MR, Castilletti C, Di Caro A, Nicastri E, Malecela MN, Ippolito G. Clinical, Virologic, and Epidemiologic Characteristics of Dengue Outbreak, Dar es Salaam, Tanzania, 2014. Emerg Infect Dis. 2016;22(5):895-9.

6. Mwanyika GO, Sindato C, Rugarabamu S, Rumisha SF, Karimuribo ED, Misinzo G, Rweyemamu MM, Abdel Hamid MM, Haider N, Vairo F, Kock R, Mboera LEG. Seroprevalence and associated risk factors of chikungunya, dengue, and Zika in eight districts in Tanzania. Int J Infect Dis. 2021;111:271-80.

7. World Health Organization. Rapid Risk Assessment of Acute Public Health Events. 2012 [cited 2024 Feb 7]. Available from: <https://www.who.int/publications/i/item/rapid-risk-assessment-of-acute-public-health-events>.

8. World Health Organization. Early warning alert and response (EWAR) in emergencies: an operational guide. 2023 [cited 2024 Mar 5]. Available from: <https://www.who.int/publications/i/item/9789240063587>.

9. European Center for Disease Prevention and Control. Operational tool on rapid risk assessment methodology. 2019 [cited 2024 Feb 7]. Available from: <https://www.ecdc.europa.eu/sites/default/files/documents/operational-tool-rapid-risk-assessment-methodolgy-ecdc-2019.pdf>.

10. Liu Y, Lillepold K, Semenza JC, Tozan Y, Quam MBM, Rocklöv J. Reviewing estimates of the basic reproduction number for dengue, Zika and chikungunya across global climate zones. Environ Res. 2020;182:109114.

11. World Bank. World Bank Open Data. 2023 [cited 2024 Feb 13]. Available from: <https://data.worldbank.org>.

12. World Health Organization. The Global Health Observatory. 2023 [cited 2024 Feb 15]. Available from: <https://www.who.int/data/gho>.

13. World Economic Forum. The Travel & Tourism Competitiveness Report 2019. 2019 [cited 2024 Feb 14]. Available from: <https://www3.weforum.org/docs/WEF_TTCR_2019.pdf>.

14. Pfeffer DA, Lucas TCD, May D, Harris J, Rozier J, Twohig KA, Dalrymple U, Guerra CA, Moyes CL, Thorn M, Nguyen M, Bhatt S, Cameron E, Weiss DJ, Howes RE, Battle KE, Gibson HS, Gething PW. malariaAtlas: an R interface to global malariometric data hosted by the Malaria Atlas Project. Malar J. 2018;17(1):352.

15. World Health Organization. Monitoring and evaluating digital health interventions: A practical guide to conducting research and assessment. 2016 [cited 2024 Feb 27]. Available from: <https://www.who.int/publications/i/item/9789241511766>.

16. Office of Public Health Data Surveillance and Technology. Dengue Virus Infections: 2015 Case Definition. 2021 [cited 2024 Jan 29]. Available from: <https://ndc.services.cdc.gov/case-definitions/dengue-virus-infections-2015/>.

17. Ministry of Health - MoH/Tanzania. Notice to the Public regarding rumors of the presence of an unknown diease in the Kagera region. 2023 [cited 2024 Feb 27]. Available from: <https://www.moh.go.tz/storage/app/uploads/public/641/54a/c73/64154ac73bc8d348261855.pdf>.

18. World Health Organization. Diesease Outbreak News: Marburg virus disease - United Republic of Tanzania. 2023 [cited 2024 Feb 27]. Available from: <https://www.who.int/emergencies/disease-outbreak-news/item/2023-DON451>.

19. Ministry of Health - MoH/Tanzania. Report of illness with symptoms of fever accompanied by hemorrhage in Ruangwa Council, Lindi Region. 2022 [cited 2024 Feb 27]. Available from: <https://www.moh.go.tz/storage/app/uploads/public/62d/546/469/62d5464696c3b531459878.pdf>.

20. World Health Organization. Diesease Outbreak News: Leptospirosis - United Republic of Tanzania. 2022 [cited 2024 Feb 27]. Available from: <https://www.who.int/emergencies/disease-outbreak-news/item/2022-DON403>.

21. World Health Organization. Weekly Bulletin on outbreaks and other emergencies in the WHO African region: Week 5, 26 January - 1 February 2019. 2019 [cited 2024 Feb 21]. Available from: <https://iris.who.int/bitstream/handle/10665/279981/OEW05-2601022019.pdf>.

22. Affara M, Lagu HI, Achol E, Karamagi R, Omari N, Ochido G, Kezakarayagwa E, Kabatesi F, Nkeshimana A, Roba A, Ndia MN, Abudo MU, Kabanda A, Mpabuka E, Mwikarago EI, Kutjok PE, Samson DD, Deng LL, Moremi N, Kelly ME, Mkama PBM, Magesa A, Balinandi SK, Pimundu G, Nabadda SN, Puradiredja DI, Hinzmann J, Duraffour S, Gabriel M, Ruge G, Loag W, Ayiko R, Sonoiya SS, May J, Katende MJ, Gehre F. The East African Community (EAC) mobile laboratory networks in Kenya, Burundi, Tanzania, Rwanda, Uganda, and South Sudan-from project implementation to outbreak response against Dengue, Ebola, COVID-19, and epidemic-prone diseases. BMC Med. 2021;19(1):160.
