# Supplementary material for: Integrated rapid risk assessment for dengue fever in settings with limited diagnostic capacity and uncertain exposure: Development of a methodological framework for Tanzania
Source: PLoS Negl Trop Dis. 2025 Mar 28;19(3):e0012946. doi: 10.1371/journal.pntd.0012946 (PMC11978086; doi:10.1371/journal.pntd.0012946)
Supplement: S1 Table — (DOCX) [file pntd.0012946.s003.docx]

**S1 Table**

**Number of confirmed dengue fever cases in 2019, by month and region (1, 2).**

*For better readability, minus is used instead of zero.*

| **Region** | **Jan** | **Feb** | **Mar** | **Apr** | **May** | **June** | **July** | **Aug** | **Sept** | **Oct** | **Nov** | **Dec** | **Total** |
| --- | --- | --- | --- | --- | --- | --- | --- | --- | --- | --- | --- | --- | --- |
| *Arusha* | - | - | - | - | - | 3 | 5 | 1 | - | - | - | - | 9 |
| *Dar es Salaam* | 25 | 67 | 227 | 1,079 | 3,093 | 1,132 | 557 | 66 | 8 | 1 | 1 | 23 | 6,279 |
| *Dodoma* | - | - | - | - | - | 3 | - | - | - | - | - | - | 3 |
| *Geita* | - | - | - | - | - | - | - | - | - | - | - | - | - |
| *Iringa* | - | - | - | - | - | - | - | - | - | - | - | - | - |
| *Kagera* | - | - | - | - | - | 2 | - | - | - | - | - | - | 2 |
| *Katavi* | - | - | - | - | - | - | - | - | - | - | - | - | - |
| *Kigoma* | - | - | - | - | - | - | - | - | - | - | - | - | - |
| *Kilimanjaro* | - | - | - | - | 1 | - | - | - | - | - | - | - | 1 |
| *Lindi* | - | - | - | - | - | 1 | 11 | 1 | - | - | - | - | 13 |
| *Manyara* | - | - | - | - | - | - | - | - | - | - | - | - | - |
| *Mara* | - | - | - | - | - | - | - | - | - | - | - | - | - |
| *Mbeya* | - | - | - | - | - | - | - | - | - | - | - | - | - |
| *Morogoro* | - | - | - | - | 6 | 15 | 1 | - | - | - | - | - | 22 |
| *Mtwara* | - | - | - | - | - | - | - | 1 | - | - | - | - | 1 |
| *Mwanza* | - | - | - | - | - | - | - | - | - | - | - | - | - |
| *Njombe* | - | - | - | - | - | - | - | - | - | - | - | - | - |
| *Pwani* | - | - | - | - | - | 38 | 63 | 6 | 1 | - | - | 2 | 110 |
| *Rukwa* | - | - | - | - | - | - | - | - | - | - | - | - | - |
| *Ruvuma* | - | - | - | - | - | - | 1 | - | - | - | - | - | 1 |
| *Shinyanga* | - | - | - | - | - | - | - | - | - | - | - | - | - |
| *Simiyu* | - | - | - | - | - | - | - | - | - | - | - | - | - |
| *Singida* | - | - | - | 1 | - | 1 | 1 | - | - | - | - | - | 3 |
| *Songwe* | - | - | - | - | - | - | - | - | - | - | - | - | - |
| *Tabora* | - | - | - | - | - | - | - | - | - | - | - | - | - |
| *Tanga* | 31 | 16 | 7 | 20 | 54 | 110 | 93 | 14 | 1 | - | - | 5 | 351 |
| *Zanzibar* | - | - | - | - | - | - | - | - | - | - | - | - | - |
| ***Total*** | 56 | 83 | 234 | 1,100 | 3,154 | 1,305 | 732 | 89 | 10 | 1 | 1 | 30 | 6,795 |

**References**

1. Mghamba J, Kauki G, Mbaga V, Moshi S, Nyanga A, Moremi N, Kakulu R, Birago J, Malima R, Mreta L, Emidi B, Adinani A, Mnyonge L, Mohammed H, Mbulumi D, Bernad J, Mwakapasa E, Camara N, Kishimba R, Gweba M, Sembuche S, Simba A, Massa K, Massaga J, Subi L, Bakari M. Tanzania Takes Measures to Stop the Spread of Dengue. Tanzania Public Health Bulletin. 2019;1(2):39-46.

2. Kauki G, Moshi S, Mwakapasa E, Kishimba R, Sembuche S, Bernad J, Simba A, Mwakapeje E, Camara N, Mmbaga V, Massaga J, Subi L, Mghamba J, Bakari M. Integrated Disease Surveillance and Response (IDSR): Cumulative report for six months, July – December 2019 (WHO weeks 27-52). Tanzania Public Health Bulletin. 2020;1(4):3-9.
