# Supplementary material for: Integrated rapid risk assessment for dengue fever in settings with limited diagnostic capacity and uncertain exposure: Development of a methodological framework for Tanzania
Source: PLoS Negl Trop Dis. 2025 Mar 28;19(3):e0012946. doi: 10.1371/journal.pntd.0012946 (PMC11978086; doi:10.1371/journal.pntd.0012946)
Supplement: S2 Table — (DOCX) [file pntd.0012946.s004.docx]

**S2 Table**

1. **Summary of contextual data indicators and their characteristics (reference period: 2019; reference administrative unit: district)**

| **Category / Risk factor** | | | | **Spatial availability** | **Temporal availability** | **Most recent** | **Quality of evidence** | **Confidence in assigning risk** | **Data source** |
| --- | --- | --- | --- | --- | --- | --- | --- | --- | --- |
| **COMPARTMENT II** | | | |  |  |  |  |  |  |
|  | ***Survival Conditions*** | | |  |  |  |  |  |  |
|  |  | | Minimum temperature | District | Daily | 2019**^‡^** | Mostly good | Good | Meteostat (1), CHIRTSdaily (2) |
|  | ***Suitability Conditions*** | | |  |  |  |  |  |  |
|  |  | | Mean temperature | District | Daily | 2019**^‡^** | Mostly good | Good | Meteostat (1), CHIRTSdaily (2) |
|  |  | | Seasonality | Country | Cross-sectional | NA | Mostly satisfactory | Unsatisfactory | Worldbank Climate Knowledge Portal (3) |
|  | ***Plausibility Conditions*** | | |  |  |  |  |  |  |
|  |  | | Urban environment / built-up index | District | Daily | 2019**^‡^** | Mostly good | Good | Copernicus Global Land Service (4) |
|  |  | | Historical dengue case reports | Regional | Cross-sectional | 2019**^‡^** | Mostly satisfactory | Satisfactory | WHO AFRO Weekly Bulletin (5), WHO EIOS (6), scientific literature |
| **COMPARTMENT III** | | | |  |  |  |  |  |  |
| ***Outbreak progression*** | | | |  |  |  |  |  |  |
|  | ***Generic*** | | |  |  |  |  |  |  |
|  |  | ***Health care capacity and access*** | |  |  |  |  |  |  |
|  |  | Medical laboratory personnel density | | Region | Annually | 2014 | Mostly good | Unsatisfactory | Tanzania National Bureau of Statistics derived from Open Data Africa (7) |
|  |  | ***Sociodemographic*** | |  |  |  |  |  |  |
|  |  | Population density | | District | Annually | 2019**^‡^** | Mostly satisfactory | Good | WorldPop/Modelling (8) |
|  |  | Household members (average) | | Region | Cross-sectional | 2017**^‡^** | Mostly good | Satisfactory | Demographic and Health Survey Program (9) |
|  |  | ***Socioeconomic*** | |  |  |  |  |  |  |
|  |  | Males’ secondary education or higher† | | Region | Cross-sectional | 2015**^‡^** | Mostly satisfactory | Unsatisfactory | Demographic and Health Survey Program (10) |
|  |  | Females’ secondary education or higher† | | Region | Cross-sectional | 2015**^‡^** | Mostly satisfactory | Unsatisfactory | Demographic and Health Survey Program (10) |
|  |  | Gini index (income inequality index) | | Region | Cross-sectional | 2017**^‡^** | Mostly satisfactory | Satisfactory | Demographic and Health Survey Program (9) |
|  |  | Less than 2.00 USD per day (poverty gap) | | District | Cross-sectional | 2010 | Mostly satisfactory | Unsatisfactory | WorldPop/Modelling (11) |
|  | ***Dengue-specific*** | | |  |  |  |  |  |  |
|  |  | ***Health care capacity and access*** | |  |  |  |  |  |  |
|  |  | Rapid diagnostic test (RDT) availability | | Country | Cross-sectional | 2019**^‡^** | Mostly good | Unsatisfactory | Scientific literature/expert knowledge: Rapid diagnostic tests have been the preferred laboratory tool for diagnosing dengue. Confirmatory tests (mainly PCR) and genomic sequencing are unlikely to be available for all samples collected during an outbreak, mainly due to resource constraints. No laboratory-based surveillance or routine diagnostic testing for DENV at most health facilities in the country (12, 13) |
|  |  | Confirmatory diagnostics, e.g., ref. lab | | Country | Cross-sectional | 2019**^‡^** | Mostly good | Unsatisfactory |  |
|  |  | ***Sociodemographic*** | |  |  |  |  |  |  |
|  |  | Population aged 15-49 (mobile pop.) | | District | Annually | 2019**^‡^** | Mostly satisfactory | Good | WorldPop/Modelling (8) |
|  |  | ***Mobility infrastructure*** | |  |  |  |  |  |  |
|  |  | Quality of airport infrastructure | | District | Cross-sectional | 2023 | Mostly satisfactory | Satisfactory | Open Street Map (Infrastructure), World Economic Forum (14) |
|  |  | Quality of seaport infrastructure | | District | Cross-sectional | 2023 | Mostly satisfactory | Satisfactory | Open Street Map (Infrastructure), World Economic Forum (14) |
|  |  | Quality of railroad infrastructure | | District | Cross-sectional | 2023 | Mostly satisfactory | Satisfactory | Open Street Map (Infrastructure), World Economic Forum (14) |
|  |  | Quality of road infrastructure | | District | Cross-sectional | 2023 | Mostly satisfactory | Satisfactory | Open Street Map (Infrastructure), World Economic Forum (14) |
|  |  | ***Water, sanitation, and hygiene*** | |  |  |  |  |  |  |
|  |  | Access to basic water infrastructure | | Region | Cross-sectional | 2017**^‡^** | Mostly good | Satisfactory | Demographic and Health Survey Program (9) |
|  |  | Access to basic sanitation | | Region | Cross-sectional | 2017**^‡^** | Mostly good | Satisfactory | Demographic and Health Survey Program (9) |
|  |  | Access to basic garbage collection | | Region | Cross-sectional | 2012 | Mostly good | Unsatisfactory | Tanzania Census 2012 (15) |
|  |  | ***Knowledge, attitudes, and behavior*** | |  |  |  |  |  |  |
|  |  | Using one Insecticide-treated Net (ITN) | | Region | Cross-sectional | 2017**^‡^** | Mostly good | Satisfactory | Demographic and Health Survey Program (9) |
|  |  | Evidence of Ae. aegypti abundance | | Country | Cross-sectional | 2019**^‡^** | Mostly satisfactory | Unsatisfactory | Scientific literature/expert knowledge: *Ae. aegypti* has been detected in several regions of Tanzania, particularly in urban environments on the east coast. Ecological models suggest conducive environmental conditions for the mosquito in the country. Recurrent outbreaks suggest a well-established mosquito population (13, 16, 17) |
|  |  | Vector control strategies available | | Country | Cross-sectional | 2017 | Mostly good | Unsatisfactory | Expert knowledge: Control measures are applied, but mostly on a small scale, primarily in urban areas or in response to an outbreak event. According to the literature, no continuous vector control measures have been introduced (18, 19) |
|  |  | ***Ecology and environment*** | |  |  |  |  |  |  |
|  |  | Urban environment / built-up index | | District | Annually | 2019**^‡^** | Mostly satisfactory | Good | WorldPop/Modelling (4) |
| ***Outbreak impact on the population and economy*** | | | |  |  |  |  |  |  |
|  | ***Generic*** | | |  |  |  |  |  |  |
|  |  | | ***Health care capacity and access*** |  |  |  |  |  |  |
|  |  | | Physician density | Region | Cross-sectional | 2014 | Mostly good | Satisfactory | Tanzania National Bureau of Statistics derived from Open Data Africa (7) |
|  |  | | Nurses and midwife density | Region | Cross-sectional | 2014 | Mostly good | Satisfactory | Tanzania National Bureau of Statistics derived from Open Data Africa (7) |
|  |  | | Hospital beds | Country | Cross-sectional | 2010 | Mostly good | Unsatisfactory | WHO Global Health Observatory (20) |
|  |  | | Healthcare accessibility (in minutes) | District | Cross-sectional | 2019 | Mostly satisfactory | Good | Malaria Atlas Project/Modelling (21) |
|  | ***Dengue-specific*** | | |  |  |  |  |  |  |
|  |  | | ***Sociodemographic*** |  |  |  |  |  |  |
|  |  | | Population aged 5-39 (morbidity relevant) | District | Annually | 2019**^‡^** | Mostly satisfactory | Good | WorldPop (8) |
|  |  | | Population aged <15 (mortality relevant) | District | Annually | 2019**^‡^** | Mostly satisfactory | Good | WorldPop (8) |
|  |  | | Population aged 65+ (mortality relevant) | District | Annually | 2019**^‡^** | Mostly satisfactory | Good | WorldPop (8) |
| **AMPLIFYING FACTORS** | | | |  |  |  |  |  |  |
|  |  | | Seasonality | Country | Cross-sectional | NA |  | Unsatisfactory | Worldbank Climate Knowledge Portal (3) |
|  |  | | Current outbreak data in the African region | Country^ƒ^ | Weekly | 2019**^‡^** | Mostly unsatisfactory | Unsatisfactory | WHO AFRO Weekly Bulletin (5), WHO EIOS (6), scientific literature |
| *^†^ Half the total weight for secondary education or higher as a risk factor;* ***^‡^*** *data also available after 2019 (including cross-sectional data collection points);* ^ƒ^ spatial details provided can vary | | | | | | | | | |

1. **Classifying “quality of evidence” and “confidence in assigning risk” informed by the ECDC’s technical report “Operational tool on rapid risk assessment methodology” (22)**

| ***Quality of evidence*** | |
| --- | --- |
| Mostly unsatisfactory | High uncertainty/conflicting data or views among experts, fragmented data, data source not verified/unknown, data collection/processing unknown |
| Mostly satisfactory | Data coming from reliable source/expert opinion, data mostly complete, data collection/processing described (incl. modelling, inter/extrapolation) |
| Mostly good | Data collected locally, data coming from one or more reliable source(s)/expert opinion(s), data mostly complete, data collection/processing described (no modelling, inter/extrapolation) |
| ***Confidence in assigning risk*** | |
| Unsatisfactory | Little/poor quality of evidence, most recent data point dates back 3+ years, data only available at the highest spatial level (national) |
| Satisfactory | Adequate quality of evidence, most recent data point dates back 1-3 years, data only available at the next higher spatial level (regional) |
| Good | Adequate/good quality of evidence, current data point available, data available at district level |

**References**

1. Meteostat. Weather Stations Python Library. 2021 [cited 2024 May 24]. Available from: <https://dev.meteostat.net/python/stations.html#example>.

2. Climate Hazard Center. CHIRTSdaily data. 2024 [cited 2024 May 24]. Available from: <https://www.chc.ucsb.edu/data/chirtsdaily>.

3. World Bank. Climate Knowlegde Portal. 2021 [cited 2024 May 24]. Available from: <https://climateknowledgeportal.worldbank.org/country/tanzania/climate-data-historical>.

4. Buchhorn M, Smets B, Bertels L, De Roo B, Lesiv M, Tsendbazar N-E. Copernicus Global Land Service: Land Cover 100m: collection 3: epoch 2015: Globe. Zenodo. 2020.

5. World Health Organization. Weekly Bulletin on outbreaks and other emergencies in the WHO African region. 2017-2024 [cited 2024 May 23]. Available from: <https://www.afro.who.int/health-topics/disease-outbreaks/outbreaks-and-other-emergencies-updates>.

6. World Health Organization. Epidemic Intelligence from Open Sources (EIOS). 2021 [cited 2024 Feb 19]. Available from: <https://www.who.int/initiatives/eios>.

7. Ministry of Health - MoH/Tanzania. Tanzania Health Facility Register. 2020 [cited 2024 Feb 26]. Available from: <https://hfrportal.moh.go.tz/index.php?r=facilities/homeAdvancedSearch>.

8. WorldPop (<www.worldpop.org> - School of Geography and Environmental Science, University of Southampton; Department of Geography and Geosciences, University of Louisville; Departement de Geographie, Universite de Namur) and Center for International Earth Science Information Network (CIESIN), Columbia University. Global High Resolution Population Denominators Project - Funded by The Bill and Melinda Gates Foundation (OPP1134076). 2018 [cited 2024 May 24]. Available from: <https://dx.doi.org/10.5258/SOTON/WP00646>.

9. Ministry of Health Community Development Gender Elderly Children - MoHCDGEC/Tanzania, Ministry of Health - MoH/Zanzibar, National Bureau of Statistics - NBS/Tanzania, Office of the Chief Government Statistian - OCGS/Zanzibar, ICF. Tanzania Malaria Indicator Survey 2017 - Final Report. 2018 [cited 2024 May 24]. Available from: <http://dhsprogram.com/pubs/pdf/MIS31/MIS31.pdf>.

10. Ministry of Health Community Development Gender Elderly Children - MoHCDGEC/Tanzania Mainland, Ministry of Health - MoH/Zanzibar, National Bureau of Statistics - NBS/Tanzania, Office of Chief Government Statistician - OCGS/Zanzibar, ICF. Tanzania Demographic and Health Survey and Malaria Indicator Survey 2015-2016. 2016 [cited 2024 May 24]. Available from: <https://dhsprogram.com/pubs/pdf/fr321/fr321.pdf>.

11. Tatem A, Gething P, Bhatt S, Weiss D, Pezzulo C. Pilot high resolution poverty maps, University of Southampton/Oxford. 2013 [cited 2024 May 24]. Available from: <https://hub.worldpop.org/doi/10.5258/SOTON/WP00290>.

12. Kelly ME, Msafiri F, Affara M, Gehre F, Moremi N, Mghamba J, Misinzo G, Thye T, Gatei W, Whistler T, Joachim A, Lema N, Santiago GA. Molecular Characterization and Phylogenetic Analysis of Dengue Fever Viruses in Three Outbreaks in Tanzania Between 2017 and 2019. PLoS Negl Trop Dis. 2023;17(4):e0011289.

13. World Health Organization. Surveillance and control of arboviral diseases in the WHO African region: assessment of country capacities. WHO; 2022 [cited 2024 May 24]. Available from: <https://www.who.int/publications/i/item/9789240052918>.

14. World Economic Forum. The Travel & Tourism Competitiveness Report 2019. 2019 [cited 2024 Feb 14]. Available from: <https://www3.weforum.org/docs/WEF_TTCR_2019.pdf>.

15. National Bureau of Statistics - NBS/Tanzania. Tanzania Census 2012. Households with access to garbage collection. 2014 [cited 2024 Feb 26]. Available from: <https://www.nbs.go.tz/index.php/en/census-surveys/population-and-housing-census/164-2012-phc-tanzania-basic-demographic-and-socio-economic-profile%20S.%20202>.

16. Philbert A, Msonga A. Dengue Vector Distribution and their Infection Status in Selected Regions in Tanzania. Tanzania Journal of Science. 2020;46(3):636-46.

17. Kraemer MUG, Reiner RC, Jr., Brady OJ, Messina JP, Gilbert M, Pigott DM, Yi D, Johnson K, Earl L, Marczak LB, Shirude S, Davis Weaver N, Bisanzio D, Perkins TA, Lai S, Lu X, Jones P, Coelho GE, Carvalho RG, Van Bortel W, Marsboom C, Hendrickx G, Schaffner F, Moore CG, Nax HH, Bengtsson L, Wetter E, Tatem AJ, Brownstein JS, Smith DL, Lambrechts L, Cauchemez S, Linard C, Faria NR, Pybus OG, Scott TW, Liu Q, Yu H, Wint GRW, Hay SI, Golding N. Past and future spread of the arbovirus vectors Aedes aegypti and Aedes albopictus. Nat Microbiol. 2019;4(5):854-63.

18. Kweka EJ, Kimaro EE, Kimaro EG, Nagagi YP, I MI. Major Disease Vectors in Tanzania: Distribution, Control and Challenges. Biological Control of Pest and Vector Insects. InTech; 2017 [cited 2024 May 23]. Available from: <http://dx.doi.org/10.5772/67109>.

19. Ngingo BL, Mboera LEG, Chengula A, Machelle I, Makange MR, Msolla M, Mwanyika GO, Rugarabamu S, Misinzo G. Aedes aegypti abundance, larval indices and risk for dengue virus transmission in Kinondoni district, Tanzania. Trop Med Health. 2022;50(1):1.

20. Ministry of Health - MoH/Tanzania. Tanzania National Health Portal. Regional Annual Health Profile 2018. 2019 [cited 2024 Feb 26]. Available from: <https://hmisportal.moh.go.tz/hmisportal/>.

21. Pfeffer DA, Lucas TCD, May D, Harris J, Rozier J, Twohig KA, Dalrymple U, Guerra CA, Moyes CL, Thorn M, Nguyen M, Bhatt S, Cameron E, Weiss DJ, Howes RE, Battle KE, Gibson HS, Gething PW. malariaAtlas: an R interface to global malariometric data hosted by the Malaria Atlas Project. Malar J. 2018;17(1):352.

22. European Center for Disease Prevention and Control. Operational tool on rapid risk assessment methodology. 2019 [cited 2024 Feb 7]. Available from: <https://www.ecdc.europa.eu/sites/default/files/documents/operational-tool-rapid-risk-assessment-methodolgy-ecdc-2019.pdf>.
