# Supplementary material for: Integrated rapid risk assessment for dengue fever in settings with limited diagnostic capacity and uncertain exposure: Development of a methodological framework for Tanzania
Source: PLoS Negl Trop Dis. 2025 Mar 28;19(3):e0012946. doi: 10.1371/journal.pntd.0012946 (PMC11978086; doi:10.1371/journal.pntd.0012946)
Supplement: S3 Table — (DOCX) [file pntd.0012946.s005.docx]

**S3 Table**

**Viral hemorrhagic fever and arboviral diseases of public health importance in Tanzania**

| **Viral hemorrhagic fever diseases** (1, 2) | **Arboviral diseases** (3) |
| --- | --- |
| Crimean-Congo hemorrhagic fever* | Chikungunya fever* |
| Dengue hemorrhagic fever* | Dengue fever* |
| Ebola virus disease | Rift valley fever* |
| Lassa fever | Yellow fever |
| Marburg virus disease* | Zika virus disease* |
| Rift Valley fever* |  |
| Yellow fever |  |

* Reported and/or presence of antibodies against the viruses across Tanzania

**References**

1. Rugarabamu S, Sindato C, Rumisha SF, Mwanyika GO, Misinzo G, Lim HY, Mboera LEG. Community knowledge, attitude and practices regarding zoonotic viral haemorrhagic fevers in five geo-ecological zones in Tanzania. BMC Health Serv Res. 2023;23(1):360.

2. Rugarabamu S, Rumisha SF, Mwanyika GO, Sindato C, Lim HY, Misinzo G, Mboera LEG. Viral haemorrhagic fevers and malaria co-infections among febrile patients seeking health care in Tanzania. Infect Dis Poverty. 2022;11(1):33.

3. World Health Organization. Surveillance and control of arboviral diseases in the WHO African region: assessment of country capacities. WHO; 2022 [cited 2024 May 24]. Available from: <https://www.who.int/publications/i/item/9789240052918>.
