# Supplementary material for: Integrated rapid risk assessment for dengue fever in settings with limited diagnostic capacity and uncertain exposure: Development of a methodological framework for Tanzania
Source: PLoS Negl Trop Dis. 2025 Mar 28;19(3):e0012946. doi: 10.1371/journal.pntd.0012946 (PMC11978086; doi:10.1371/journal.pntd.0012946)
Supplement: S4 Table — (DOCX) [file pntd.0012946.s006.docx]

**S4 Table**

**Glossary**

*Types of surveillance to monitor infectious diseases of public health concern (1-3)*

(i) Scope of data collection approaches and target groups/sites (selection)

| **Surveillance strategy** | **Description** |
| --- | --- |
| Active surveillance | Proactively collect health data, such as from public health authorities, healthcare providers, and medical records to determine the prevalence of a specific disease or health condition using ​standardized definitions and predetermined criteria. |
| Passive surveillance | Regular and systematic reporting of diseases and health conditions by health professionals in the population and in health facilities in a given area; health reports are referenced and analyzed by public health authorities responsible for data management, completeness, and reliability. |
| Sentinel surveillance | Focuses on one or a small number of surveillance sites (health facilities) that collect data on a disease or health condition in the population using standardized definitions and predefined criteria; sentinel sites do not have a predefined catchment area/population; useful for collecting epidemiologic information such as affected groups, risk factors, monitoring hospitalizations, data collection for epidemiologic studies, etc. |
| Population-based surveillance | Collection of data on a disease or health condition in the population in a specific geographic area (catchment area) by appropriate healthcare facilities, using standardized definitions and predefined criteria; data may be representative of the country (national) or on subnational level (e.g., regions, districts); useful for determining disease rates, such as incidence and mortality rates. |
| Indicator-based surveillance | Systematic collection, monitoring, analysis, and interpretation of structured data about a disease or health condition from healthcare facilities or other appropriate sources, using standardized definitions and predefined criteria. |
| Event-based surveillance | Manual or automated near/real-time collection and monitoring of unstructured, ad-hoc data and information (including unverified) about potential health events or risks of public health significance; data sources may include medical and non-medical information sources, such as news reports or social media. |
| Early Warning, Alert and Response (EWAR/S) | Organized mechanisms or system to rapidly detect and promptly respond to signals indicating an acute public health risk or threat. |

(ii) Scope of the surveillance priorities (selection)

| **Surveillance strategy** | **Description** |
| --- | --- |
| Demographic surveillance | Longitudinal collection of data and statistics on population demographics, such as data on births, deaths, and marital status, for a dynamic cohort representative of the total population in a predefined area. |
| Syndromic surveillance (including clinical and health-related data) | Real-time collection, monitoring, analysis, and interpretation of non-specific health-related data (e.g., clinical signs, symptoms) that support early detection of a potential health event; may also include medication prescription data, chief complaint data, absenteeism, and other data related to an outbreak; high sensitivity due to lack of laboratory confirmation. |
| Laboratory-confirmed surveillance | Collect and analyze data on laboratory-confirmed cases of a disease or health condition to monitor the etiologic agent, detect changes early, and inform control measures (e.g., vaccine composition, drug resistance). |
| Serosurveillance  (Laboratory-confirmed) | Collection and analysis of blood samples to determine disease burden or immunity levels/gaps in the population; usually conducted as a periodic survey to monitor immunity trends over time. |
| Genomic surveillance (Laboratory-confirmed) | Collection and analysis of pathogen genome sequence data from representative populations to monitor pathogen trends, identify genetic similarities and differences, and detect new variants. |
| Zoonotic surveillance/ Animal health | Collecting, monitoring, analyzing and interpreting data on animal health events, e.g., mass mortality events, with a special focus on zoonotic agents, by veterinarians and animal health authorities. |
| Zoonotic surveillance/  Vector distribution | Collect, monitor, analyze, and interpret entomological and snail distribution data to determine health risks and inform vector control efforts. |
| Environmental (health) surveillance | Collecting, monitoring, analyzing, and interpreting data on environmental hazards or precipitating factors, using environmental samples (e.g., water, soil, air) or monitoring of environmental changes (e.g., toxic pollution, temperature conditions, access to clean water). |
| Biosurveillance | Collect, monitor, analyze, and interpret data from human and animal populations and the environment to detect and identify biological threats, including (bio)terrorist threats. |

*What else to know when reading our manuscript (4, 5) :-)*

| **Term** | **Description** |
| --- | --- |
| Climate change | The process of changing from one state of regional or global climate to another. It is preferred to "global warming" because it conveys that the effects include more than just rising temperatures. |
| Ecology | The scientific discipline under consideration pertains to the examination of the interactions between organisms and their physical environment. |
| Ecosystem | A functional unit consists of all the living organisms in a given area, along with the non-living components of the environment, such as air, soil, water, and sunlight. It is studied to understand how changes affect the interactions between these organisms and their environment. |
| Endemic | The baseline level of disease usually present. |
| Epidemic | The occurrence of more cases of a disease than expected in a particular area or among a particular group of people over a period of time. |
| Outbreak | Unexpected increase in the incidence of a specific disease within a specific time period and geographic area, which refers to either an epidemic or a pandemic. |
| Pandemic | An epidemic that occurs over a very large area (several countries or continents) and usually affects a large proportion of the population. |
| R0, basic reproduction number | It indicates the average number of secondary infections produced by an infected individual in a fully susceptible population. |
| Risk assessment | A systematic process for assessing the likelihood and potential impact of an infectious disease outbreak. |
| Signal | A "signal" is an alert or indicator that indicates a potential increase in disease activity or an emerging outbreak. |
| Vector | An organism (usually an arthropod like flea, mosquito, or tick) that transmits a pathogen from one host to another. |

**References**

1. World Health Organization. Early warning alert and response (EWAR) in emergencies: an operational guide. 2023 [cited 2024 Mar 5]. Available from: <https://www.who.int/publications/i/item/9789240063587>.

2. Murray J, Cohen A. Infectious disease Surveillance. In: International Encyclopedia of Public Health. Elsevier, 2017: 222-9.

3. Abat C, Chaudet H, Rolain JM, Colson P, Raoult D. Traditional and syndromic surveillance of infectious diseases and pathogens. Int J Infect Dis. 2016;48:22-8.

4. The National Academy of Sciences. Infectious Disease - Glossary. 2025 [cited 2025 Jan 15]. Available from: <http://needtoknow.nas.edu/id/glossary/>.

5. Centers for Disease Control and Prevention (CDC). Epidemiology Glossary. 2025 [cited 2025 Jan 15]. Available from: <https://www.cdc.gov/reproductive-health/glossary/index.html#cdc_generic_section_10-p>.
