# Supplementary material for: Integrated rapid risk assessment for dengue fever in settings with limited diagnostic capacity and uncertain exposure: Development of a methodological framework for Tanzania
Source: PLoS Negl Trop Dis. 2025 Mar 28;19(3):e0012946. doi: 10.1371/journal.pntd.0012946 (PMC11978086; doi:10.1371/journal.pntd.0012946)
Supplement: Attachment — Submitted filename: Response-to-reviewers.pdf [file pntd.0012946.s009.pdf]

January 15, 2025

Editorial Board

PLOS NTD

Dear Rebecca C. Christofferson, Victoria Brookes, Shaden Kamhawi, and Paul Brindley,

dear Editors,

I wish to resubmit the manuscript entitled "**Integrated Rapid Risk Assessment for Dengue Fever in Settings with Limited Diagnostic Capacity and Uncertain Exposure: Findings from a Pilot Study in Tanzania**" The Manuscript ID is PNTD-D-24-00913.

We would like to express our gratitude to you and the reviewers for your constructive feedback. Following a thorough review of the manuscript, the necessary modifications have been implemented in accordance with the provided recommendations. The rationale behind the signal refinement algorithm (Compartment II) has been strengthened, and some minor adaptations have been made throughout the manuscript. Our responses to all comments have been prepared and are provided below.

Thank you for your consideration. I look forward to hearing from you.

Sincerely,

Matthias Belau, Dr.PH

Reviewer 1 Comments

Please find below a point-by-point response to all comments (in italics). All page, line, and table numbers refer to the revised manuscript, where changes are highlighted.

METHODS:

(1) The reviewer commented, "The aim of the study or goal of the study is mentioned. The objectives (small definitions of goals) in a stepwise manner can be written more clearly. The study design is very complex and, as such, not clearly mentioned; rather, described as frameworks or compartments, which may take a lot of time or are sometimes not able to be understood by readers."

Response: *>>> We've added some extra info to the last paragraph of the Introduction to make things clearer, including minor goals mentioned there (on page 5, lines 116ff). We really hope this helps! This paper is all about how we developed our framework and how we assessed its conceptual feasibility, hence, it's not a traditional study design (i.e., cohort study), which might make it a bit different for some readers. We really believe that this format and journal are the best way to communicate rapid risk assessment for dengue fever to diverse readers, especially in countries with limited diagnostic capacity and uncertain exposure. Please tell us exactly what you didn't understand so that we can improve it.*

(2) The reviewer commented, "The figure 2 of context driven factors should take into account the presence of vector mosquitoes, any entomological surveillance or indicators. The other factors like previous cases and temperature alone may be not be considered sufficient to draw a framework for dengue risk assessment which depends so much on day biting vector and people's habits of storing water or recreational activities like fountains, waterpots, and the artificial collection of water in containers (container index for mosquito surveillance)...in lines 176 to 193 the investigators have themselves described the importance of vector habits and breeding patterns in dengue risk assessment, but temperature and rainy patterns alone may not decide "survival and suitability" factors in the study"

Response: *>>> We fully agree with the reviewer that combining entomological data, including vector control strategies, and event-based surveillance can create a more effective framework for identifying and managing dengue outbreaks. However, our working hypothesis is that in resource-restricted settings, like Tanzania, it is not proven nor realistic to have routine surveillance capacities in place, like vector infestation indices or routine sampling and testing of mosquitoes year-round, referring to our introduction (page 5, lines 106ff). For Compartment II, we're focusing on refining clinical signals indicating suspected cases using contextual information. Therefore, our main concern is not the distribution of the vector (including whether it is highly or rarely distributed) but the likelihood of its presence indicated by environmental conditions when it comes to quickly assessing a syndrome for plausibility. We did a comprehensive literature review that indicated temperature and precipitation are the most important covariates for the Aedes life cycle and persisting dengue transmission. If you want to know more about our methodological approach, please refer to page 8, lines 181ff in the methods, or see the Supplementary Material S1 for more details.*

(3) The reviewer commented, "I find this factor plus integrated vector management efforts and surveillance factors consideration missing in this manuscript. What about cases that are diagnosed timely and not diagnosed? They should be taken into account and mentioned clearly in simple language in a summary or in conclusions as considered suitable by authors."

Response: *>>> We appreciate your comment and kindly refer to our response to comment #2. We designed our methodological framework tailored to the context of resource-restricted settings, where routine surveillance isn't necessarily established and effective vector monitoring and control might not be in place or is lacking (openly available) reporting. As described in Figure 1 (page 7) and outlined on page 12, lines 254ff, if cases have been confirmed by laboratory diagnostics (see Table 3, page 13), we do integrate them in our risk assessment framework. For cases that haven't been diagnosed but present symptoms according to our case definition, outlined on page 7, Table 1, we assess them for plausibility to be caused by dengue virus, which you can find under Compartment II. A statement on the limitations of this approach and, after all, highlighting the importance of adequate diagnostic and integrated surveillance capacity has been added on page 23, line 492ff. Additionally, in the assessment of each case, we emphasize the importance of clarifying diagnostics (see Table 6 on page 17 'Suggested action points').*

(4) The reviewer commented, "The sample size sufficiency cannot be determined as existing WHO guidelines are compared against the author's work. As I understand it, there is only one author involved in data collection from one region of the continent. Unless multiple foci involving different health systems and surveillance are involved, the data or findings cannot be generalised."

Response: *>>> Unfortunately, we need additional information to properly address your concern. We laid out our methodological framework in detail, highlighting its scalability to different geographic settings upon contextual adaptation. While this allows flexible implementation, it certainly limits generalizability. To test its conceptual feasibility and showcase its strengths and limitations, we used openly available data to perform a risk assessment for Tanzania, and we validated both Compartment II and Compartment III against published event-based surveillance data (for Compartment II, we used WHO EIOS data to identify signals of suspected dengue cases, and for Compartment III, we relied on registry data reported from Tanzania via WHO reports). The data represents the number of reported confirmed dengue cases in 2019, stratified by month and region. No primary data has been collected through the associated project "ESIDA" for feasibility testing and validation. Given the limited project resources and the risk of missing positive cases in an extensive field data collection for testing purposes, this was the most feasible approach.*

(5) The reviewer commented, "The statistical analysis is described separately for different compartments. The quantity and quality of information in this article are exhausting for understanding, and sometimes figures and tables are not relatable to each other."

Response: *>>>. We are aware that this is a complex framework, but we believe that it follows a common thread in its sequence and methodological approach. Again, we kindly ask you to be more explicit about what is hard to understand, or which figure and table have no relationship.*

(6) The reviewer commented, "statistical tests used are not described; only framework for defining risk assessment is given. The basic definitions are not given. The authors should consider giving the existing terms and definitions used by researchers all over the world."

Response: *>>> We've described the statistical analyses (regression analyses) in the methods, so you can find them under the subheading "Statistical analyses" (see page 16, lines 332ff). We're still a little confused about what's meant by "basic definitions are not given. The authors should consider giving the existing terms and definitions [authors' comment: of what?] used by researchers all over the world". We'd really love to understand this better so we can make any improvements needed.*

(7) **The reviewer commented, “This seems like a secondary data study (infectious disease modelling) maintaining name-wise anonymity, but region- and site-wise description and risk assessment are not anonymous.”**

Response: *>>> Thank you for this observation. We'd like to clarify that our approach focuses on aggregate signal processing, for example, using WHO EIOS as one potential data source. Our methodology intentionally operates at a population level, analyzing trends in suspected infection numbers rather than individual-level data. This aligns with the core purpose of an early warning framework, where aggregate signals are sufficient for detecting emerging patterns. However, if we've misunderstood your concern about anonymity, we welcome further clarification.*

(8) **The reviewer commented, “The data collection and validation guidelines can be more specific, depending on author's preferences and objectives of the study”**

Response: *>>> We put together a summary of all secondary data inputs for Compartment II and Compartment III to make things easier for you and the readers. This includes information on each datasets source, the time of collection/publication and an assessment of its quality in the context of our study. You can find this overview in the Supplementary Material, in section S4. Any details on the test signal data (derived from openly available reports and WHO EIOS) for the Compartment II assessment, you can find in Table 5 (page 15). The number of confirmed dengue fever cases in 2019, broken down by month and region, is outlined in a table with references in the Supplementary Material. For easier navigation, you can find all this in the methods section under the heading "Data availability" (see page 15, lines 322).*

(9) **The reviewer commented, “The methods section can benefit by a table of definitions of frequently used terms in this study, eg; hazard ratio, R0, signals, risk assessment, early warning surveillance, EBS, survival signals, etc. The readers will find it easier to follow the results and conclusions with the help of table of terms and acronyms .”**

Response: *>>> Thank you for this suggestion for improvement. We have added a glossary to the Supplementary Materials to make our work easier to follow and to help readers better understand less common terms. We would like to note that the comment lists some terms that we did not use in our work. These terms are therefore not included in the glossary.*

(10) **The reviewer commented, “In Line 219, "expert discussions," please specify and give references here. The associated details for "compartment III" are in supplementary file. A small footnote accompanying the table and explaining the columns of the table and risk level scoring is desirable in the methods.”**

Response: *>>> We have now listed the areas of expertise of the experts involved in the discussion (see page 10, lines 232f). They all were members of the ESIDA network on which this work is based (see Acknowledgements). They are all listed as authors of the manuscript. A detailed description of the columns and the risk level scoring is given in the body text after the table (see page 12, lines 245ff).*

RESULTS:

(11) **The reviewer commented, “The whole work is comprehensive and complex. If the authors consider presenting it in more parts, more readers, researchers and care providers will be able to understand and follow their message. Authors are to congratulated on their efforts for this work and submit it in few words. I think a book or chapterwise explanation may do more justice to this project. The actual data will need presentation. This MS describes methods and framework. More work and visual depiction of reaching results is**

needed to be actually incorporated in the MS. Maybe two parts of article that how framework was designed by reviewing the data and how risk assessment for Tanzania was done. A statistical analyst may comment more on results and Chloropeth charts with review of supplementary files. Figures are clear. The results need more clarity and comparisons."

Response: *>>> We chose PLOS NTD as our publication venue because it is an established open-access journal that effectively reaches our target audience and has a strong track record in disseminating novel conceptual frameworks and research findings. Our paper primarily focuses on the development of our conceptual framework, using dengue in Tanzania as a case simulation due to its global relevance and the specific characteristics of our study site. In a next step, we are keen to pilot our framework under real-world conditions and are eager to receive feedback on its practicality from public health stakeholders. We plan to investigate this aspect in future research and will share those findings in subsequent publication accordingly. As for statistical analysis, we would like to note that our analytical approach and visualization methods were developed and thoroughly reviewed by our research team, including our senior epidemiologist and biostatistician (last author). However, if you have specific concerns about the statistical analysis or choropleth maps that you would like us to address, we welcome your detailed feedback.*

CONCLUSIONS:

(12) The reviewer commented, "The methods is novel and designed with appropriate reviews and integration but how it was utilised in field and actual situation is not clear from results and conclusions."

Response: *>>> We would like to refer to our response to comments #1 and #11. The present manuscript delineates the methodological evolution of the framework with specific reference to dengue and Tanzania. A validation in the field under real-time conditions remains to be conducted.*

EDITORIAL AND DATA PRESENTATION MODIFICATIONS?

(13) The reviewer commented, "I would like to express my gratitude to the Journals Editorial Team for giving me this opportunity to review this article. The amount of work done and statistical modelling and concepts are novel, though somewhat larger in quantity and quality. Average readers and researchers can find this article difficult to understand and apply in their settings. The content has to be read again and again for understanding the concept. Moreover, how these new risk assessment strategies may benefit community or health program implementation needs more context and clarity."

Response: *>>> We believe that the study and its results are relevant to the scope of PLOS NTD and will be of interest to its readership though it is indeed of high complexity. This paper describes how we developed a novel yet feasible framework for integrated rapid risk assessment, with a focus on dengue in Tanzania while taking into account data constraints. To reduce complexity, we divided the different assessment steps into compartments and illustrated them through case simulations. Figure 4 additionally aims to demonstrate what final information is prepared for the decision-maker (made as accessible and simple as possible), while highlighting that the data integration, processing, and analytical concept of such approaches are non-trivial. We, therefore, appreciate your interest in the implementation phase. Following our conceptual feasibility testing, we plan to field-test this approach through potential public health partnerships, e.g., with the East African Community (EAC) and/or Tanzania's Ministry of Health (MoH), to enable real-time operation. As outlined in our conclusions, we have identified key stakeholders who would derive significant value from this*

frameworks's implementation (see page 25, lines 541ff). Therefore, we share your enthusiasm for advancing this work toward practical application.

(14) The reviewer commented, "I have another query in my mind: Usually in any situation, we recommend strengthening diagnosis and surveillance; then should we not focus more on strategies to improve surveillance rather than preparing newer frameworks based on assumptions and definitions and existing criteria?"

Response: *>>> We do not intend to suggest that frameworks like ours can replace robust diagnostics and surveillance. Rather, our aim is to complement these by integrating contextual data and supporting initial assessments when rapid action is required but diagnostics are not always readily and specifically available - particularly in resource-limited settings. For this reason, we emphasize on page 22, line 474-477, that our framework can inform and coordinate diagnostic capacities, such as mobile laboratories, in a targeted manner, integrating epidemiological and ecological concepts as well as event-based surveillance and outbreak signal detection. In addition, we highlighted the importance of complementing diagnostic and surveillance capacity rather than replacing them on page 22, line 466ff.*

SUMMARY AND GENERAL COMMENTS

(15) The reviewer commented, "The article is written to suggest a new integrated framework for dengue risk assessment in context-specific settings of Tanzania in east Africa. The authors state that existing WHO Epidemic Intelligence from Open Source Systems (EMIOS), e.g., WHO EWRS for dengue, requires continuous surveillance and laboratory services networks. These ideal situations of diagnosis and surveillance are not present in Tanznaia and other LMIC settings, and there is a need for event-based surveillance (EBS)."

Response: *>>> Thank you for this summary, which supports our response to comment #14. We believe that innovative approaches utilizing early outbreak signals and contextual data, which are typically available more quickly than laboratory diagnostics, offer particular value for resource-restricted settings. Through our work, which places special emphasis on scalability and openly available data, we aim to highlight this potential.*

(16) The reviewer commented, "The title says- Limited Diagnostic Capacity and Uncertain Exposure: Findings from a Pilot Study. What were criteria to decide that the diagnostic capacities were limited and exposure was uncertain?"

Response: *>>> To develop this work, we worked closely with our colleagues from the Nelson Mandela African Institution in Arusha, Tanzania, as well as representatives of the Mobile Laboratory activities of the East African Community. In addition, our analysis draws on both field experience and literature review. Tanzania faces significant public health system challenges, as reflected in its Global Health Security Index assessment of prevention, detection, and response capacities (https://ghsindex.org/country/tanzania/). The significance of dengue as a public health concern in Tanzania is demonstrated by the extensive 2019 outbreak and reported increasing outbreak activity in East African countries since 2023 specifically, also highlighting existing surveillance and response limitations.*

(17) The reviewer commented, "The work is comprehensive but very difficult to understand. Is this compilation of several articles for determing risk assessment or analysis of cases from Tanzania or as stated in introduction report of recent outbreak."

Response: *>>> Our paper presents a methodological framework for risk assessment, which we developed, illustrated and tested for conceptual feasibility using dengue in Tanzania as a case study. To enhance clarity, we have addressed all points raised and added a glossary to the Supplementary Materials. We welcome specific feedback on any sections that may require further clarification.*

(18) The reviewer commented, "Real- world data—WHO and Tanzania. (how is that collected and compared) and in what manner integrated with existing EWS; should be written clearly."

Response: *>>> Our framework incorporates real-world data from two primary sources: WHO EIOS signals of suspected dengue cases and official dengue case registry data. The WHO EIOS (Epidemic Intelligence from Open Sources) system, described in detail at https://www.who.int/initiatives/eios/eios-technology, provides a standardized approach to early signal detection. In our manuscript, we demonstrate how these data sources can be systematically integrated into existing early warning systems, with the process exemplified across three case studies in the results section. This integration approach prioritizes practical applicability while maintaining methodological rigor.*

(19) The reviewer commented, "In what ways is the discussed- "integrated surveillance assessment system" NOVEL from existing approaches"

Response: *>>> We appreciate the opportunity to clarify the novel aspects of our framework. Our approach offers several key innovations:*

(1) Our framework uniquely integrates rapid risk assessment with early warning by combining real-time signal detection and routine health reports with contextual vulnerability and capacity data - a feature not present in existing systems to the best of our knowledge
(2) We specifically designed the framework to be implementable in resource-limited settings by prioritizing openly available data sources and scalable methods
(3) Unlike existing approaches that often require extensive local data infrastructure, our framework adapts to different data availability scenarios while maintaining systematic assessment capabilities

These distinctions from current systems are detailed on page 5, line 98-111 as well as throughout the discussion section and summarized in the conclusion section (page 25, line 536-541) of the manuscript, where we compare our framework to existing approaches.

We appreciate your careful reading of our manuscript and the constructive comment. We have tried to answer your questions to the best of our ability.

Reviewer 2 Comments

Please find below a point-by-point response to all comments (in italics). All page, line, and table numbers refer to the revised manuscript, where changes are highlighted.

METHODS:

(1) The reviewer commented, "The study is of great interest when considering the rise of arboviruses on the African continent, where many countries have an insufficient level of preparedness. Predicting dengue outbreak risk is particularly valuable to anticipate and manage future outbreaks. The objectives are clearly stated, and the study design aligns with the hypothesis being tested. While the methodology is generally well-conceived, I am critical of Compartment 2: Refinement of Clinical Signals Using Contextual Data. I disagree with some of the contextual factors used for refinement:
- The life history data of Aedes aegypti lacks references for development time and lifespan. The latter, which may be underestimated, requires references to be credible.
- The key factors identified for Aedes aegypti include temperature, relative humidity, and rainfall, which may be correlated in pairs."

Response: >>> *We appreciate the critical review of Compartment II. While we acknowledge that our assessment is simplified, our framework deliberately relies on indicators that can be serviced through openly available data, particularly temperature and rainfall (humidity is not consistently available as an open dataset from all weather stations or satellite data). We based our selection of covariates on the most thoroughly researched factors in the scientific literature, prioritizing implementability in our framework design. We acknowledge these inherent limitations, but given that our primary aim is to conduct plausibility checks of symptomatic, unconfirmed cases - often under significant time constraints - we believe this approach remains feasible. We welcome suggestions for expanding the critical discussion beyond what is currently presented. To address the concern about temporal parameters, we have expanded and clarified our description of the relevant timeframes (see page 8, lines 181ff). We have also included literature on developmental time and lifespan. There are a few studies under laboratory conditions that report longer adult lifespans when evaluating the effect of diet on longevity (e.g., https://doi.org/10.1590/1678-4162-12080). It is also true that meteorological data such as temperature, humidity and rainfall are correlated. We deliberately chose seasonality as a proxy for humidity and rainfall. In the rainy season, the probability of the vector occurring is higher, while in the dry season, when temperatures are also lower on average, the probability of the vector occurring is lower. This interaction between the individual factors is also taken into account in our algorithm (Compartment II).*

RESULTS:

(2) The reviewer commented, "The analysis presented matches the initial plan, and the results are clearly presented. The discussion does not overlook the results. The tables and figures are sufficiently illustrative of the findings."

Response: >>> *We appreciate the reviewer's positive assessment of our manuscript's structure, clarity, and alignment between analysis plan and results presentation.*

CONCLUSIONS:

(3) **The reviewer commented, “The study addresses the important topic of dengue risk prediction and provides a framework for risk assessment in Tanzania that could be applied to other countries.”**

Response: *>>> We thank the reviewer for recognizing the broader applicability of our framework beyond the Tanzanian case study, which aligns with our goal of developing a scalable approach for dengue risk assessment.*

SUMMARY AND GENERAL COMMENTS

(4) **The reviewer commented, “The study is of great interest when considering the rise of arboviruses on the African continent. By developing a framework of dengue risk prediction for Tanzania, the authors provide tools to develop models for other African countries where dengue is on the rise.”**

Response: *>>> We thank the reviewer for highlighting the broader relevance of our work in the context of emerging arboviruses across Africa. This observation aligns with our intention to develop an adaptable framework that can support public health decision-making beyond Tanzania.*

We thank the reviewer for the thoroughly reading of our manuscript and the constructive comments. We have tried to answer your questions to the best of our ability.

Editorial Comments

Please find below a point-by-point response to all comments (in italics). All page, line, and table numbers refer to the revised manuscript, where changes are highlighted.

(1) The editorial commented, "Please ensure that the CRediT author contributions listed for every co-author are completed accurately and in full. (…) Please ensure that the full contributions of each author are acknowledged in the "Add/Edit/Remove Authors" section of our submission form."

Response: *>>> We have revised this item to explicitly list each author accurately and in full. We hope it is clearer.*

(2) The editorial commented, "Please upload all main figures as separate Figure files in .tif or .eps format."

Response: *>>> We now provide all images in tagged image file format (tiff) at 600dpi each.*

(3) The editorial commented, "We have noticed that you have uploaded Supporting Information files, but you have not included a list of legends. Please add a full list of legends for your Supporting Information files after the references list."

Response: *>>> We now provide a complete list of legends for our supporting information files after the reference list.*

(4) The editorial commented, "Some material included in your submission may be copyrighted. (…) Potential Copyright Issues:
i) Figures 3, and 4. Please (a) provide a direct link to the base layer of the map (i.e., the country or region border shape) and ensure this is also included in the figure legend; and (b) provide a link to the terms of use / license information for the base layer image or shapefile."

Response: *>>> The shapefile for Tanzania Districts (administrative level 2), which was used for creating this work, was originally accessed through https://www.nbs.go.tz/index.php/en/census-surveys/gis/568-tanzania-districts-shapefiles-2019 (currently only accessible via Wayback Machine: https://web.archive.org/web/20240522075041/https://www.nbs.go.tz/index.php/en/census-surveys/gis/568-tanzania-districts-shapefiles-2019, last accessible 2024-05-22). The same shapefile is currently accessible via https://www.nbs.go.tz/statistics/topic/gis (last accessed: 2025-01-08). Source is National Bureau of Statistics in Tanzania. License Statement: "This agreement allows the permitted user a worldwide royalty-free non-exclusive licence to freely use the data, copy, modify, translate, publish, adapt, distribute, create derivative works and value-added products for commercial and non-commercial purposes subject to the terms of this licence." The license statement ist available under https://www.nbs.go.tz/nbs/takwimu/references/Licence-Agreement-NBS.pdf (last accessed: 2025-01-08)."*

(5) The editorial commented, "Please amend your detailed Financial Disclosure statement. This is published with the article. It must therefore be completed in full sentences and contain the exact wording you wish to be published.
1) State the initials, alongside each funding source, of each author to receive each grant. For example: "This work was supported by the National Institutes of Health (####### to

AM; ###### to CJ) and the National Science Foundation (###### to AM).".
If you did not receive any funding for this study, please simply state: u201cThe authors received no specific funding for this work.u201d”

Response: *>>> We have placed the detailed financial statement before the references (see page 27, lines 595ff).*

We thank the editor for the thoroughly reading of our manuscript and the constructive comments. We have tried to answer your questions to the best of our ability.
