# Supplementary material for: Integrated rapid risk assessment for dengue fever in settings with limited diagnostic capacity and uncertain exposure: Development of a methodological framework for Tanzania
Source: PLoS Negl Trop Dis. 2025 Mar 28;19(3):e0012946. doi: 10.1371/journal.pntd.0012946 (PMC11978086; doi:10.1371/journal.pntd.0012946)
Supplement: Attachment — Submitted filename: Response.docx [file pntd.0012946.s010.docx]

February 19, 2025

Editorial Board

**PLOS NTD**

Dear Rebecca C. Christofferson, Victoria Brookes, Shaden Kamhawi, and Paul Brindley,
dear Editors,

I would like to resubmit the manuscript entitled "**Integrated Rapid Risk Assessment for Dengue Fever in Settings with Limited Diagnostic Capacity and Uncertain Exposure: Development of a Methodological Framework for Tanzania**" with the manuscript ID is PNTD-D-24-00913R1.

We thank you and the reviewers for your constructive feedback. In line with the recommendations provided, we have made changes to the title and discussed how the framework fills existing gaps and how it could be integrated into existing systems in Tanzania. Our responses to all comments have been prepared and are provided below.

Thank you very much for your consideration. I look forward to hearing from you.

Sincerely,

Matthias Belau, Dr.PH

**Editorial Comments**

Please find below a point-by-point response to all comments (in italics). All page, line, and table numbers refer to the revised manuscript, where changes are highlighted.

(1) **The editorial commented, “The title could be changed to better reflect... something like: "Development of a methodological framework for ...." would be a better representation rather than "findings from a pilot study" which infers a more data research study. This study is a framework validation, as presented.”**

**Response:** *>>> We have changed the title as suggested.*

(2) **The editorial commented, “In the discussion, there could be more discussion on how this framework fill existing gaps and how it could be integrated into existing systems - such as MoH in Tanzania.”**

**Response:** *>>> We have revised the discussion as suggested, focusing more specifically on existing gaps and the integration of our framework into existing systems in Tanzania (please see pages 22ff).*

Once again, we thank the editor for the thorough review of our manuscript and the constructive comments. We have tried to answer your questions to the best of our ability.
